# Engineered Cell-Based Therapeutics: Synthetic Biology Meets Immunology

**DOI:** 10.3389/fbioe.2019.00043

**Published:** 2019-03-18

**Authors:** Fabio Caliendo, Marina Dukhinova, Velia Siciliano

**Affiliations:** ^1^Istituto Italiano di Tecnologia-IIT, Largo Barsanti e Matteucci, Naples, Italy; ^2^Imperial College London, South Kensington, London, United Kingdom

**Keywords:** synthetic biology, immunology, T cell engineering, synthetic immunology, engineered cell-based therapy

## Abstract

Synthetic Biology has enabled new approaches to several medical applications including the development of immunotherapies based on bioengineered cells, and most notably the engineering of T-cells with tumor-targeting receptors, the Chimeric Antigen Receptor (CAR)-T cells. CAR-T-cells have successfully treated blood tumors such as large B-cell lymphoma and promise a new scenario of therapeutic interventions also for solid tumors. Learning the lesson from CAR-T cells, we can foster the reprogramming of T lymphocytes with enhanced survival and functional activity in depressing tumor microenvironment, or to challenge diseases such as infections, autoimmune and chronic inflammatory disorders. This review will focus on the most updated bioengineering approaches to increase control, and safety of T-cell activity and to immunomodulate the extracellular microenvironment to augment immune responses. We will also discuss on applications beyond cancer treatment with implications toward the understanding and cure of a broader range of diseases by means of mammalian cells engineering.

## Introduction

Synthetic biology is particularly suited for the development of safer immune-therapeutics by means of genetic reprogramming of immune cells (Kitada et al., 2018; Tokarew et al., 2018), or other mammalian cells to confer immune-mimetic characteristics (Liu et al., 2018). Genetically engineered circuits are based on the assembly of well characterized biological parts with a predictable behavior (di Bernardo et al., 2012; Menolascina et al., 2012; Wieland and Fussenegger, 2012), providing insights into dynamics of network topologies (Tigges et al., 2009; Siciliano et al., 2011; di Bernardo et al., 2012) and uncovering new roles of biomolecules (Siciliano, 2013).

Synthetic devices with therapeutic applications rely on *smart interfaces* that is the assembly of sensing and actuating modules with high bio-computing capabilities. By implementing Boolean logic gates, genetic circuits integrate multiple extracellular and intracellular inputs, achieving sophisticated control of timing, localization, specificity, and strength of transgene expression, outsmarting classical cell engineering approaches typically based on ON-OFF switches. Sensor modules use intracellular (e.g., microRNA, proteins; Xie et al., 2011; Wroblewska et al., 2015; Siciliano et al., 2018) or extracellular (e.g., soluble molecules or surface proteins) inputs to reshape cellular fate (Kipniss et al., 2017; Scheller et al., 2018), whereas actuator modules rely on transcriptional (Stanton et al., 2014; Li et al., 2015; MacDonald and Siciliano, 2017), or translational regulation (Wroblewska et al., 2015; Cella et al., 2018). Synthetic biology can facilitate the reprograming of T-cells in a predictable and safer manner, by using safety switches and linking specific input sensing to gene expression induction (Roybal et al., 2016a; Nissim et al., 2017; Siciliano et al., 2018), potentially overcoming side effects such as on-tumor-off target effects and over-activation.

As recently T-cell engineering has extensively overviewed (Marshall and Djamgoz, 2018; Si et al., 2018; Tokarew et al., 2018), here we review recent developments of synthetic biology-based strategies to improve efficacy, specificity, and strength of current T-cell therapies, mostly advanced in blood and tumor malignancies. We will also discuss the design of synthetic devices to treat viral or bacterial infections that are implemented in different subpopulation of T-cells or other mammalian cells which are then conferred of immune-mimetic characteristics.

### Chronicles of T Cell Engineering

T lymphocytes are critical components of adaptive immunity and participate in immune responses against disorders including cancer, viral and bacterial infections, autoimmune conditions, and chronic inflammation. Cytotoxic CD8^+^ T-cells express the T-cell receptor (TCR) that recognize epitopes displayed by MHC class I molecules on the surface of almost every cell in the body. CD8^+^ T-cells activated upon antigen recognition directly kill target cells through the release of cytotoxic granules.

Engineered cell-based immunotherapies, were initially based on the integration of exogenous T-cell receptor (TCR) in cancer patient's autologous T-cells. The TCR is a heterodimeric protein consisting of variable α and β chain, associated with invariable dimeric signaling molecules: CD3 δ/ε, CD3 γ/ε, CD247 ζ/ζ. The variable chains recognize the antigen expressed on the surface of target cells whereas the invariable CD3 chains propagate the signal. This is further strengthened by the simultaneous binding of the co-receptor (in proximity of TCR) to MHC molecule on the surface of target cells. This approach used to target MART-1 melanoma antigen, resulted in regression of metastatic melanoma in 13% of patients after adoptive T-cell transfer (Morgan et al., 2006), but the non-perfect matching between exogenous TCR and HLA molecules of the recipient limited its efficacy.

**Figure 1:**
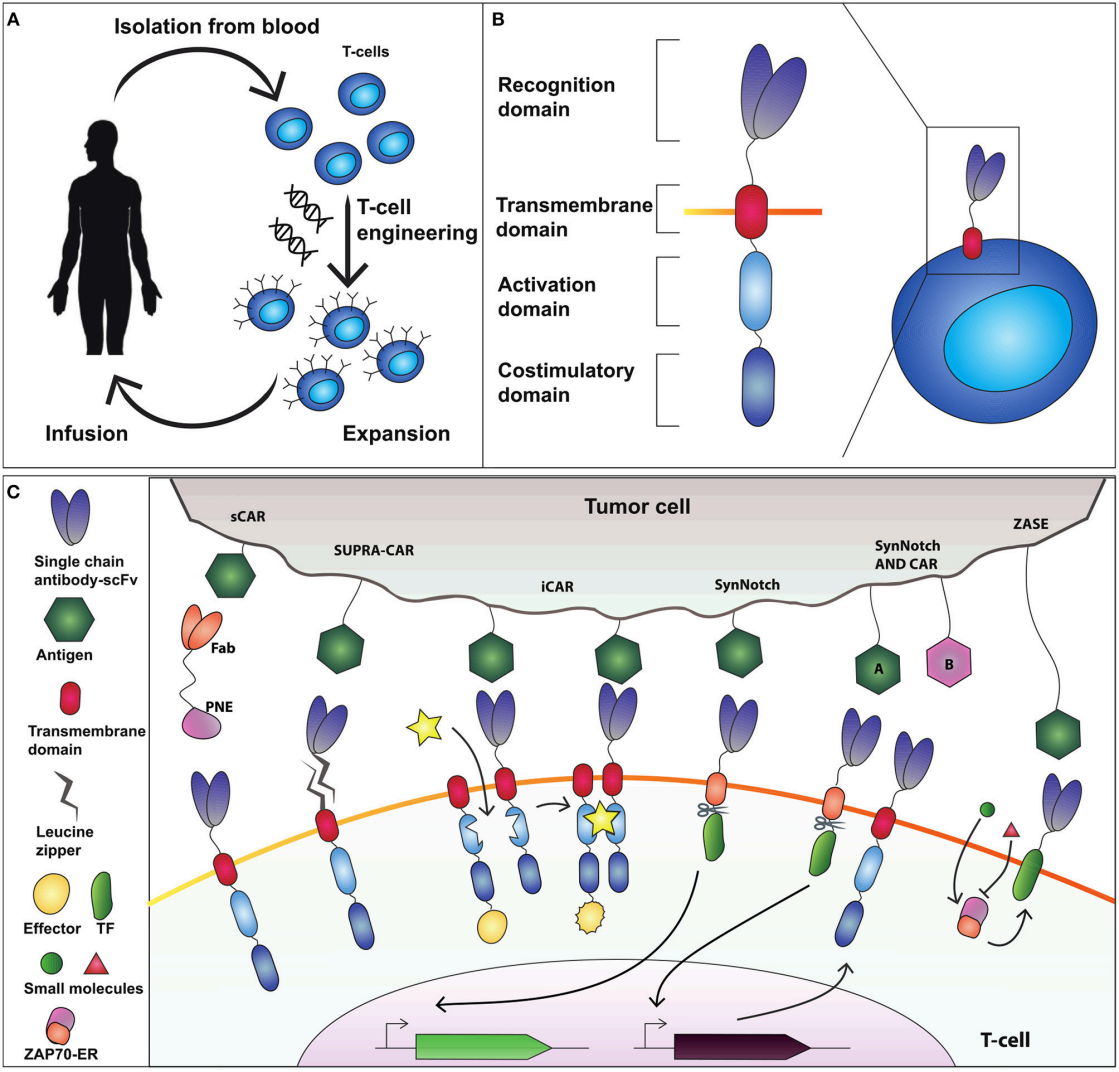
Adoptive T-cell therapy and chimeric antigen receptor (CAR) design. **(A)** T-cells are isolated from the blood and genetically modified to express a cancer-targeting receptor. Engineered T cells are then expanded and transfused back into the patient. **(B)** The current CAR design comprises an extracellular recognition domain (a single chain fragment antibody-scFv for binding to the antigen) a transmembrane domain needed to anchor the receptor to the cell membrane, an activation domain (CD3ζ chain) and a costimulatory domain (commonly CD28 and 4-1BB). **(C)** Synthetic devices to improve specificity of CAR-T-cell activity. The sCAR system includes a switchable CAR (sCAR) and a Fab fragment fused to a yeast-derived peptide neo-epitope (PNE_tag). The Fab domain of the Fab-PNE module recognizes a tumor-associated antigen (TAA) whereas the sCAR specifically binds the PNE domain of the switch. This interaction activates sCAR-T cells against tumor cells. SUPRA-CAR is a two-component system consisting of a universal domain CARs expressing a leucin zipper extracellular domain (zipCAR) and a tumor-targeting scFv adapator (zipFv). The scFv of zipFv recognize the TAA while the zip binds the leucine zipper on the zipCAR acting as a switch. Also here it creates an immunological synapse that drives T-cell activation. iCAR consists of two incomplete CAR molecules that heterodimerize in presence of a small molecule which functions as a switch to activate T-cell response. SynNotch receptor comprises a scFv extracellular domain that recognizes the antigen, fused to a cleavable intracellular domain that retain a transcription factor (TF) to the membrane. The antigen recognition induces the cleavage of TF that translocate into the nucleus to activate output transcription. SynNotch/CAR AND-gate circuit to trigger activation when two distinct antigens are expressed on the target cell. SynNotch receptor activated by antigen “A” drives the expression of a CAR molecule which targets antigen “B.” Interaction with antigen B leads to T-cell activation. ZASE is a dual-gated ZAP70 protein switch, obtained by fusing Zap70 to the ligand-binding domain of the estrogen receptor ER^t2^. Two distinct small molecules, 4OHT (green circle) and 3-MB-PP1 (red triangle) turn, respectively ON and OFF protein activity.

Adoptive T-cell therapy using chimeric antigen receptors (CARs) obtained greater clinical success than TCR for engineered T-cell-based cancer immunotherapy (Harris and Kranz, 2016; Figure 1A). CAR-T-cells represent the most relevant synthetic biology-inspired therapeutic, and the most clinical advanced T-cell engineering platform to fight hematologic malignancies. CARs consist of an extracellular single chain fragment domain (scFv), that recognize the desired antigen fused to the intracellular activating (CD3ζ) domain and costimulatory (CD28 and 4-1BB) domains (Sadelain et al., 2017; Figure 1B). CARs can recognize different antigens molecules, and activation is MHC independent (Chmielewski et al., 2013).

Recently, CAR-T therapies Axicabtagene ciloleucel and Tisagenlecleucel were approved by the US Food and Drug Administration and the European Medicines Agency for the treatment of large B-cell lymphoma and B-cell acute lymphoblastic leukemia. They both consist of engineered autologous anti-CD19 CD8^+^ T-cells targeting B-cells, but Axicabtagene ciloeucel uses the CD28 costimulatory domain, whereas Tisagenlecleucel adopts 4-1BB. The choice of costimulatory domain has clinical impact on overall response rate (ORR) and associated toxicity including the cytokine release syndrome (CRS). Patients treated with Axicabtagene ciloleucel, showed ORR in 82% and CRS in 93% of the cases (Neelapu et al., 2017). In contrast, patients with diffuse large B-cell lymphoma treated with Tisagenlecleucel, showed ORR in 53% and CRS in 58% of the cases (Schuster et al., 2017). CRS is triggered by massive secretion of pro-inflammatory cytokines potentially leading to fatal side effects (Barrett et al., 2014; Davila et al., 2014) and is thus detrimental to broad applications of CAR-T therapy. Strategies to control T-cell activation are therefore a major goal of synthetic biology.

### Improving Safety of T Cell-Based Therapies With Synthetic Biology Approaches

State-of-the-art methods to enhancing CAR-T-cell safety include the use of drug-controlled kill switches that can eliminate CAR-T-cells in case of severe toxicity (Straathof et al., 2005; Stasi et al., 2011). However, kill switches do not provide control over T-cell activation and expansion, and the elimination of potentially therapeutic CAR-T-cells is irreversible.

Rodgers and colleagues (Rodgers et al., 2016) constructed a peptide-specific switchable CAR-T-cells (sCARs) that improve tumor-specific activation in a reversible manner. sCARs system relies on CAR-T-cells engineered with a receptor that recognize a yeast-derived peptide neo-epitope tag (PNE). The PNE is fused to antibody fragments (Fab) specific to the tumor associated antigen-TAA. Thus, Fab-PNE functions as a bridge that create immunological synapses between the tumor cells and sCAR (Figure 1C). This strategy provides a potential universal platform that can be repurposed to the selected tumor by rewiring the Fab domain fused to PNE. In addition, as the Fab fragment has a shorter half-life than a whole antibody, this system allows precise temporal control over cytokines released by sCAR-T-cells. Low doses of switch favor the generation and maintenance of a large memory T-cell populations in Nalm-6 mice cancer model, which correlates with sustained remission in hematologic malignancies (Li et al., 2013). The sCAR approach was effective also to target ubiquitous antigens overexpressed in solid tumors such as HER2 in pancreatic cancer cells, reporting low toxicity in a xenograft PDAC mouse model (Raj et al., 2018).

A further step toward the development of flexible and controllable CAR-T-cells is the split, universal and programmable chimeric antigen receptor system (SUPRA-CARs) (Cho et al., 2018).

In SUPRA-CARs, T-cells integrate a leucin zipper domain in the extracellular portion of the receptor (zipCAR), whereas the scFv specific to TAA is fused to a second leucin zipper (zipFv) that binds the cognate one on zipCAR (Figure 1C). Like sCAR approach also here zipFv operates a bridge to form immunological synapses that triggers T-cell activation. By modulating zipFv and leucine zippers affinity as well as zipCAR expression levels, it is possible to tune T-cell activation. Of note, SUPRA-CARs offer the unique possibility to activate different signaling domains and T-cell subsets (i.e., CD4^+^ and CD8^+^) in a modular fashion using orthogonal (that is non-cross-interacting), leucine zipper combinations. These systems share the potential to establish a universal treatment of several malignancies by rewiring the TAA-specific scFvs.

A third approach to achieve spatial and temporal control over engineered T-cells is the inducible CAR system (iCAR) that consists of two incomplete CAR molecules that heterodimerize in presence of a small molecule which functions as a switch to activate T-cell response (Figure 1C; Wu et al., 2015). Here, one iCAR includes the TAA-specific scFv domain fused to a heterodimerizing domain. The second iCAR contains the T-cell receptor activating the signaling domain, fused to the heterodimerizing domain. To enable precise activation of T cell response the authors used two very well-known heterodimerizing domains, namely FK506 binding protein (FKBP) domain and the T2089L mutant of FKBP-rapamycin binding domain (FRB^*^) that heterodimerize in the presence of the rapamycin analog AP21967. This ON-switch enables titratable pharmacologic regulation allowing precise control of timing, location, and dosage of T-cell activity.

A further way to control T-cells activity is to modulate Zeta-chain-associated protein kinase 70 (Zap70), a tyrosine kinase that upon TCR engagement starts downstream activation signaling. This strategy was envisioned by Wong and colleagues (Wong and Wong, 2018) that created a dual-gated ZAP70 protein switch, by fusing Zap70 to the ligand-binding domain of the estrogen receptor ER^t2^ (ZASE). The system exhibits swift temporal control using two distinct small molecules, 4-hydroxy-tamoxifen (4OHT) and 3-MB-PP1, to turn ON, and OFF protein activity, respectively (Figure 1C). This is a promising tool for the rapid and fine control of the T-cell activation in a time scale of minutes. However, two main drawbacks emerge. First, it fails to modulate cytokine release. Second, the ON switch (induction by 4OHT) is highly leaky in CARs making it difficult to use for CAR-based therapy.

### Synthetic Receptors That Activate Genetic Transcriptional Programs in T-Cells

Differently from CARs, SynNotch receptors do not activate endogenous signaling pathways, but are coupled to synthetic transcriptional systems. SynNotch receptor comprises a scFv extracellular domain that recognizes a desired epitope, fused to a user-defined cleavable intracellular domain that retain a transcription factor (TF) to the membrane. The antigen recognition induces the cleavage of TF that translocate into the nucleus to activate output expression (Figure 1C; Morsut et al., 2016). SynNotch receptors are the ideal example of a synthetic biology tool, because of their modularity, orthogonality (no cross-activation of genetic pathways) and predictability, and have been used in cancer immunotherapy to engineer the secretion of bispecific antibodies directed against cancer cells (Roybal et al., 2016b), or to achieve combinatorial antigen sensing circuits (Roybal et al., 2016a). Here, CAR-T cells were engineered with an AND-gate circuit to trigger activation toward cells expressing two distinct antigens. Specifically, the SynNotch receptor activated by antigen “A” drives the expression of a CAR molecule which targets antigen “B” (Figure 1C). Thus, the contemporary presence of two antigens will activate the T-cell response, enabling an effective target cell-specific control system. Because of their extreme flexibility, multiple synNotch receptors are ideal modules of genetic smart interfaces: they can be used in the same cell to achieve combinatorial integration of environmental cues, including Boolean response programs, multi-cellular signaling cascades, and self-organized cellular patterns.

### Synthetic Biology for Solid Tumor Treatment With T-Cell-Based Immunotherapy

CAR-T-cell therapy for solid tumors is limited by several issues. First, tumor microenvironment is hypoxic, poorly vascularized, and the extracellular matrix isolate tumors from normal tissues preventing lymphocytes access to the tumor site (Vignali and Kallikourdis, 2017). Second, inhibitory surface proteins, cytokines or soluble molecules of cell metabolism within the tumor can affect activation and persistence of T cells (Tokarew et al., 2018). Third, although clinical trials showed that CAR-T-cells can treat solid malignancies such as HER2 positive sarcomas, malignant pleural mesotheliomas, glioblastoma, non-small cell lung cancer, liver metastasis, and melanoma (Ahmed et al., 2015; Brown et al., 2015; Johnson et al., 2015; Katz et al., 2015; Feng et al., 2016; Beatty et al., 2018; Yu et al., 2018), fatal cases of off-target activity and infiltrates of T-cells in heart tissues were reported (Johnson and June, 2017).

Synthetic circuits may play a pivotal role in both enhancing tumor-killing specificity of T-cells and to skew the tumor microenvironment in favor of an improved anti-tumor T-cell response.

As previously discussed, one way to increase efficacy of T-cell-based immunotherapy for solid tumors is to augment tumor-recognition specificity and avoid CAR-T-cell self-toxicity, through the development of combinatorial antigen sensing systems. To increase specificity, an option is to use CARs that trigger antitumor functions after engagement with multiple targets present only in the pathogenic microenvironment. Zhang et al. (2018) implemented a dual-CAR (dCAR) modified T-lymphocyte to treat pancreatic cancer. They took advantage of the co-expression of two TAAs, carcino-embryonic antigen (CEA) and mesothelin (MSLN) on AsPC-1pancreatic cancer cells, to generate dCARs containing two physically separate CEA-CD3ζ and MSLN-4/1BB signaling domains controlled with CEA and MSLN, respectively. The authors obtained significant cytotoxicity for two antigen-positive tumor cells and no cytotoxicity for single antigen-positive tumor cells, alleviating “on-target, off-tumor” toxicity and enabling accurate application of CAR-T-cell therapy.

Approaches to redirect an immunosuppressive microenvironment toward an immune-stimulatory one, often use rewiring of cytokine signaling pathways. Kunert et al. (2018) engineered T cells with melanoma-specific TCR and murine IL-12 and/or IL-18 under nuclear-factor of activated T cell (NFAT) responsive promoter. Adoptive transfer of T-cells with a TCR and inducible IL-12 to melanoma-bearing mice resulted in severe toxicity with high levels of IFNγ and TNFα in blood, whereas T-cell activation upon interaction with melanoma cells promoted IL-18-mediated cytotoxic response against tumor without side effects, enhancing the presence of T-cells within tumors, and reducing tumor burden.

Another interesting attempt to protect T cells from the immunosuppressive cytokines in the tumor microenvironment is the use of synthetic autocrine loops.

Golumba-Nagy and colleagues identified that CAR-T cells with the CD28 costimulatory domain were resistant to TGF-β-mediated immunosuppression in the tumor microenvironment (Koehler et al., 2007; Golumba-Nagy et al., 2018). Further investigations showed that CD28 mediates the LCK phosphorylation which in turn promotes IL-2 secretion, a fundamental pro-survival cytokine (Golumba-Nagy et al., 2018). However, since IL-2 released by CAR-T-cells negatively impacts the anti-tumor activity through sustaining survival and function of T_reg_ cells, the authors engineered a hybrid receptor, fusing the extracellular IL-7α receptor subunit to the intracellular IL-2β receptor subunit (IL7Rα/IL2Rβ) which converted the IL-7 signal into IL-2 specific response. Together, the CAR equipped with IL7Rα/IL2Rβ hybrid receptor form a synthetic autocrine loop, that makes CAR-T-cells resistant to TGF-β-mediated suppression.

**Figure 2:**
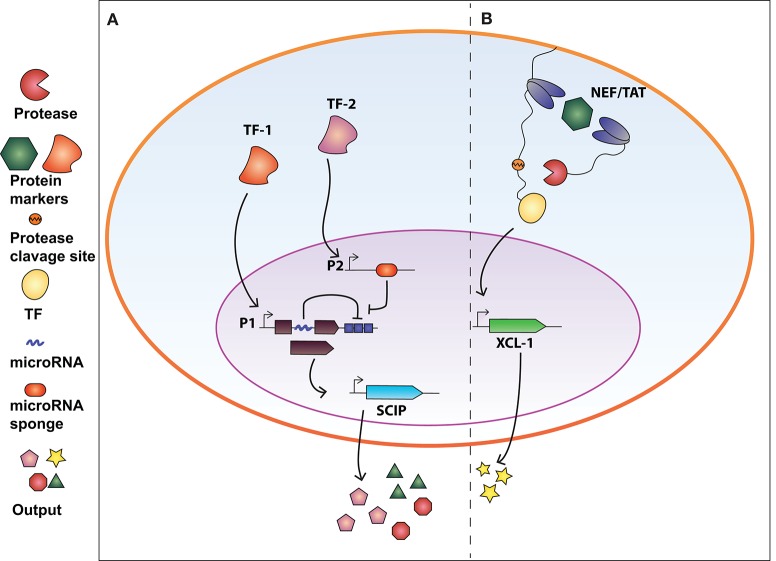
Synthetic biology devices for immunomodulation of the extracellular environment in response to intracellular protein biomarkers. **(A)** AND gate circuit activated by tumor-specific transcription factors TF-1 and TF-2. TF-1 regulates the transcription of GAD transcription factor which in turn activate the production of SCIP (cell surface antigen EST, CCL21 chemokine, IL-12, and anti-PD1 antibody). The open reading frame of GAD is divided in two exons and an intron processed into a miRNA that inhibits the GAD-encoding mRNA (negative auto-regulatory feedback). TF-2 promotes the expression of a miRNA-sponge sequesters miRNA-x allowing the expression of GAD. Therefore, both TF-1 and TF-2 are required to exert immunomodulatory regulation. **(B)** Intrabody-based device for activation of transcriptional programs in presence of a target protein. Cytosolic proteins are identified by two intracellular antibodies, one membrane-tethered and fused to a transcription factor (TF) via a TEV protease cleavage site, and the other fused to the viral TEV protease. The presence of the target protein and subsequent binding of the two intrabodies results in TEVp cleavage of TCS and release of TF, converting protein detection into transcriptional output. The system was used to engineer CD4^+^ T-cells to release XCL-1 chemokine upon detection of the HIV-1 NEF protein.

Synthetic circuits that release immune-stimulatory signals such as chemokines, cytokines, and immune checkpoint inhibitors directly in the tumor microenvironment, can attract immune cells at the tumor site. In this regard, Nissim et al. (2017) engineered an AND gate circuit activated when two distinct tumor-specific transcription factors (tTFs) are present. The device comprises two genetic modules responsive to the tTFs (Figure 2A). The input promoter1 (P1) responsive to tTF1 regulates the transcription of the synthetic transcription factor GAD. The open reading frame of GAD was divided in two exons spanned by an intron processed into a mature miRNA (miRNA-x) after splicing. miRNA-x binds to the 3′UTR of the GAD-encoding mRNA (negative auto-regulatory feedback). The input promoter2 (P2) responsive to tTF2 regulates the transcription of a miRNA sponge specific for miRNA-x. Therefore, when both tTFs are present, the miRNA-sponge sequesters miRNA-x allowing the expression of GAD, which in turn activates an immunomodulatory output called SCIP. SCIP consists of a cell surface antigen (EST), the CCL21 chemokine that facilitates T-cell migration at the tumor site, IL-12 and anti-PD1 antibody. The authors showed that the circuit effectively boost the immune antitumor response both *in vitro* and *in vivo* settings.

### Beyond Tumor Treatment: Engineering Approaches to Viral Infections

Although a huge effort has been devoted to tumor therapies, T-cell engineering may provide alternative treatments also to other diseases including viral or bacterial infections.

Haran et al. (2018) engineered CAR-T cells with CXCR5 receptor along with an all-rhesus variant of the CD4–MBL CAR to target SIV-infected T-follicular-helper-cells (Tfh) located in B-cell follicles where viral replication concentrate. CXCR5 mediates T-cell migration, whereas mannose-binding lectin (MBL) binds to the dense oligo-mannose patch that is highly conserved on clinically relevant HIV-1 variants. The authors showed potent suppression of two SIV strains *in vitro* and concentration in B-cell follicles in tissues *ex vivo*, paving the way to potential long-term durable remission of SIV and HIV infections.

In our recent work (Siciliano et al., 2018) we developed a modular cellular intrabody-based platform that detects intracellular proteins serving as diseases' biomarkers. Cytosolic proteins are identified by two intracellular antibodies, one membrane-tethered and fused to a transcription factor (TF) via a TEV protease cleavage site (TCS), and the other fused to the viral TEV protease (TEVp) (Figure 2B). The presence of the target protein and subsequent binding of the two intrabodies results in TEVp cleavage of TCS and release of TF, converting protein detection into transcriptional output. We tailored the device to detect HCV and HIV infection by targeting NS3 and Tat and NEF proteins, respectively. We also demonstrated that CD4^+^ T-cells engineered with the genetic network were able to release XCL-1 chemokines and to interfere with HIV-1-mediated downregulation of HLA molecules.

## Conclusion and Outlook

T-cell engineering represents a novel promising direction of immunotherapy of various disorders including and beyond blood and solid tumors. The most developed area of T-cell-based immunotherapy application is cancer. By designing smart interfaces that process multiple input to activate specific genetic programs, synthetic biology may enable a new wave of smart adoptive T-cell therapy, addressing current issues and enhancing efficacy and safety of tumor treatments. Several efforts have shown alternative strategies to achieve control and specificity of T-cell activation, including multiple-antigen recognition to activate T-cell response, small molecule-driven ON switches, and protein-tags for the creation of immunological synapses. Efforts focus also on immunomodulation of tumor microenvironment, to make cancer cells more susceptible to T-cell action. While CD8^+^ T-cells are the target of genetic modifications for cancer treatment, engineering of other T-cell populations may be of help to cure other disorders such as viral infections. For example, CD4^+^ T-cells have been successfully modified to respond to SIV or HIV-1 infections (Haran et al., 2018; Siciliano et al., 2018). These platforms can be useful also to monitor dynamic changes in the intra/extra-cellular environment, allowing studies of spatiotemporal characteristics of infection events and to induce localized release of therapeutic agents.

Of note, approaches that involve the engineering of other mammalian cells to confer immunocompetent characteristics are being explored. In a recent work from Liu et al. (2018) HEK293 cells were engineered with a synthetic bacteria-responsive signaling cascade that contains human Toll-like receptors as bacterial sensor and a synthetic promoter driving expression of lysostaphin, a bacteriolytic enzyme highly lethal to *Staphylococcus aureus*. Similarly, Kojima et al. (2018) built T-cell-receptor-like signal transduction devices for sensing specific cell contacts in HEK-293T cells and human MSCs (hMSCs) with potential application in cancer therapy. The synthetic biology artillery is thus continuing to grow, to face new challenges and to bring solutions to unmet medical needs. In perspective, these devices can augment the understanding of the complexity of other diseases such as immune disorders including T-cell exhaustion that prevent long-term efficacy of T-cell activity in chronic infections, inflammation, and cancer (Wherry and Kurachi, 2015), or autoimmune diseases like type-1 diabetes where autoreactive T lymphocytes trigger progressive inflammation and tissue degeneration (Bluestone et al., 2015).

These challenges will bring synthetic biology further closer to patients' needs.

## Author Contributions

FC and MD contributed equally to manuscript preparation. FC and MD wrote the manuscript. VS wrote and edited the manuscript.

### Conflict of Interest Statement

The authors declare that the research was conducted in the absence of any commercial or financial relationships that could be construed as a potential conflict of interest.
